# The role of kelp crabs as consumers in bull kelp forests—evidence from laboratory feeding trials and field enclosures

**DOI:** 10.7717/peerj.3372

**Published:** 2017-05-25

**Authors:** Katie Dobkowski

**Affiliations:** 1 Department of Biology, University of Washington, Seattle, WA, United States of America; 2 Friday Harbor Laboratories, University of Washington, Friday Harbor, WA, United States of America

**Keywords:** *Nereocystis luetkeana*, Bull kelp, *Pugettia gracilis*, Choice feeding experiments, *Pugettia producta*, Herbivory

## Abstract

The Northern kelp crab (*Pugettia producta*) and the graceful kelp crab (*Pugettia gracilis*) are common primary consumers in bull kelp beds near the San Juan Islands (Salish Sea, NE Pacific). In this system, urchins (often considered the most voracious herbivores exerting top-down control on kelp beds) tend to remain sedentary because of the high availability of detrital macroalgae, but the extent to which kelp crabs consume kelp (and other food options) is largely unknown. I conducted four types of laboratory feeding experiments to evaluate kelp crab feeding patterns: (1) feeding electivity between bull kelp (*Nereocystis luetkeana*) and seven species of co-occurring local macroalgae; (2) feeding electivity on aged vs. fresh bull kelp; (3) feeding preference between *N. luetkeana* and small snails (*Lacuna* sp.); and (4) scaling of feeding rate with body size in *P. producta* and *P. gracilis*. In choice experiments, *P. producta* consumed greater mass of *N. luetkeana* than of other macroalgal species offered and elected to eat fresh bull kelp over aged. However, *P. producta* also consumed snails (*Lacuna* sp.), indicating more generalized feeding than previously suspected. Feeding rates for *P. producta* exceeded the expected 3∕4 scaling rule of metabolic rates, indicating that larger *P. producta* may have a disproportionately large impact on bull kelp. A subtidal field experiment, designed to assess the influence of consumers on juvenile bull kelp net tissue gain, found that only fully enclosed (protected) bull kelp increased in wet mass and blade length. Herbivory by kelp crabs, among other consumers, is likely to play a previously unrecognized role in mediating the growth and survival of this annual kelp species within the Salish Sea.

## Introduction

Kelp forests provide habitat for many organisms (Steneck et al., 2002) and consumers of kelp, including various mollusks, sea urchins, and vertebrates such as odacid fishes, can strongly influence kelp distribution and abundance (Paine & Vadas, 1969; Andrew & Jones, 1990; Paine, 1992). While snails consume less biomass directly than sea urchins, damage from their grazing on blades indirectly increases tissue loss, especially during storms (Krumhansel & Scheibling, 2011). In Alaska, California, and Nova Scotia, grazing by sea urchins is a dominant top down control when urchins are present in high densities (Pearse & Hines, 1979; Duggins, 1980; Estes & Duggins, 1995; Scheibling, Hennigar & Balch, 1999). In extreme cases, sea urchins may lead to the collapse of kelp-dominated ecosystems and establishment of urchin barrens as an alternative stable state (Filbee-Dexter & Scheibling, 2014).

While sea urchins are known to destructively graze kelp beds (Leighton, 1966; Foreman, 1977), gastropods have subtler, but still important effects on kelp forests. For example, the snail *Lacuna vincta* lives and feeds on bull kelp, *Nereocystis luetkeana* (Phaeophyceae, Laminariales) in the eastern Pacific Ocean including the Salish Sea. Grazers with scraping radulae, like *L. vincta*, can influence population distribution of bull kelp by weakening the thallus (Chenelot & Konar, 2007; Duggins, Eckman & Siddon, 2001), which may be especially detrimental to juvenile bull kelp because of their small size, suggesting a need for further investigation into the role of non-echinoid grazers across bull kelp life history stages.

In addition to urchins and snails, other consumers that may affect the distribution and abundance of kelp include amphipods and herbivorous fish. High numbers of grazing amphipods after an El Nino event decimated algal biomass in the Point Loma area of southern California, decreasing drift macroalgal availability, which further resulted in destructive grazing of standing giant kelp (*Macrocystis pyrifera*) beds by sea urchins (Tegner & Dayton, 1991). In the Mediterranean Sea, the herbivorous fish *Sarpa salpa* can consume large amounts of the dominant genus of macroalga, *Cystoseira* in shallow rocky subtidal areas. Herbivorous fish drive seaweeds in this genus into deeper or more wave exposed environments or may lead to the evolution of chemical defenses, as in *C. balearica* (Vergés, Alcoverro & Ballesteros, 2009).

Decapod crustacean grazers live in kelp beds, but their effect on kelp species in different geographical regions is not entirely clear. In California, where there is no *N. luetkeana*, kelp crabs eat several of the dominant kelps: *M. pyrifera*, *Egregia menziesii* and *Pterygophora californica* (Knudsen, 1964; Leighton, 1966). In this system, the Northern Kelp crab, *Pugettia producta,* influences biomass and nutrient uptake of the feather boa kelp, *Egregia menziesii* (Bracken & Stachowicz, 2007). In central California, the preferred habitat of *P*. *producta* (and also primary food source) changes ontogenetically, with the red juveniles living among and feeding on red intertidal macroalgae, while adult kelp crabs dwell within kelp beds, and consume kelp (Hultgren & Stachowicz, 2010). Field observations as well as gut content analysis from crabs collected in central California suggests that *P. producta* lives on and specializes in feeding on *M. pyrifera* (Hines, 1982). These diverse feeding choices suggest variability in the dietary composition of kelp crabs.

Bull kelp (*N. luetkeana)* and giant kelp (*M. pyrifera*) both range from Alaska to California. However, it is *N. luetkeana*, not *M. pyrifera*, that dominates the kelp forests of the Salish Sea, including the waters surrounding the San Juan Islands of Washington state, providing food and habitat for a variety of marine species (Dayton, 1985; Steneck et al., 2002; Carney et al., 2005; Springer et al., 2006). This large kelp provides abundant food, including detrital material, to food webs within and below the photic zone (Duggins & Eckman, 1994), as well as habitat and nursery space for a variety of fish species (Carr, 1991). Red urchins playa smaller role in structuring these bull kelp forests than in many other habitats because of the high availability of drift kelp that limits their need to search for food (Britton-Simmons, Foley & Okamoto, 2009; Lowe et al., 2014).

The Northern kelp crab (*Pugettia producta*) and the graceful kelp crab (*Pugettia gracilis*) are both common in kelp beds in the Pacific Northwest, but to what degree they feed on the dominant canopy-forming kelp (*N. luetkeana*), as compared to other macroalgal, invertebrate, or detrital food choices, remains less well-studied. Kelp crab feeding patterns have not been thoroughly quantified in the lab or in the field. In this study, I combine laboratory feeding experiments with a field experiment to quantify kelp crab feeding patterns and evaluate their role as consumers. Specifically, I: (1) determine feeding choices of *P. producta* among different macroalgal species; (2) evaluate whether *P. producta* are herbivorous, detritivorous, or omnivorous; (3) quantify how food consumption scales with body size in two common species of kelp crabs (*P. producta, P. gracilis*); and (4) determine the effect, in the field, of exposure to and exclusion of kelp crabs (and other large consumers) on juvenile bull kelp net tissue gain.

## Methods

### Laboratory feeding experiments

To evaluate kelp crab feeding patterns, I used four types of laboratory feeding experiments: (1) feeding electivity between *N. luetkeana* and seven species of co-occurring macroalgae in four separate choice experiments; (2) feeding electivity on aged vs. fresh *N. luetkeana*; (3) feeding preference between *N. luetkeana* and a local snail (*Lacuna* sp.); and (4) scaling of crab body size with feeding rates on *N. luetkeana*. Studies of feeding preferences require experiments in which potential diet items are offered both separately and together to determine whether feeding rates are similar on alternative food items in both cases. Determining feeding rate involves enclosing consumers with a single food source; feeding electivity, as defined by Singer (2000), emerges from buffet-style experiments in which consumers have a choice of what to eat (Grason & Miner, 2012). I employed electivity experiments to distinguish among macroalgae common in the local environment, including the invasive brown alga, *Sargassum muticum*, addressed the possibility of a detritivore role by offering both fresh and aged *N. luetkeana,* and offered snails as a test of omnivory.

I conducted feeding experiments in 43 cm × 30 cm × 18 cm (15.1 L) plastic aquaria in a seawater table with flow-through seawater at Friday Harbor Laboratories (FHL), Washington, USA. Aquaria were covered with plastic egg crate material to prevent crabs from escaping while allowing air and water flow (∼3 L/min). I collected the organisms shortly preceding laboratory experiments by snorkeling or using SCUBA at sites near FHL and in the San Juan Channel (48°43′42.14″N, 123°00′44.02″W). Crabs and snails were maintained in flow-through aquaria and fed *ad libitum* on a mixed diet of macroalgae prior to use in experiments. A new cohort of crabs was used for each of the different feeding experiments. I starved kelp crabs for 12 h prior to experiments to reduce variability in consumption due to recent feeding history.

All data analyses were conducted with R v. 3.3.2 (R Core Team, 2016). A Shapiro–Wilk test was used to assess the normality of all residuals prior to analysis and a Levene’s test was used to test for homogeneity of variance. Due to the high levels of variability inherent to feeding experiments, the data did not meet these assumptions, and I used non-parametric statistical tests as presented in the following methods for each separate type of experiment.

#### Feeding electivity on N. luetkeana vs. co-occurring macroalgae

To determine electivity of kelp crabs for co-occurring macroalgae, I conducted four separate feeding experiments, each time offering the kelp crabs a choice of *N. luetkeana* and two other locally abundant macroalgae with varied morphology and ecological roles. Species offered in addition to *N. luetkeana* included three subtidal kelps (*Costaria costata, Saccharina latissima, Agarum fimbriatum*), an intertidal kelp (*Alaria marginata*), one invasive brown alga (*S. muticum*), as well as one red alga (*Mazzaella splendens*) and one green macroalga (*Ulva* sp.). These subtidal species tested represent two different functional groups of native seaweeds and one invasive seaweed that occurs in the San Juan Islands; the subtidal kelps form the canopy structure below *N. luetkeana*, while the smaller red and green seaweeds offered belong to the understory (Britton-Simmons, 2006). I included the intertidal kelp *A. marginata* because kelp crabs sometimes reside in intertidal zones and would likely encounter this species. I included *N. luetkeana* in each of the experiments because crabs consistently chose to eat it in pilot studies. I offered macroalgae in groups of three to suit the size of the experimental arenas that I used.

Before each experiment, I wiped the macroalgal thalli clean with a paper towel to reduce associated bacteria and epiphytes. Pieces were approximately 10 cm × 30 cm, with some variation due to algal morphology. This standardized size ensured that some macroalgae remained at the end of the feeding experiment for measurement (eliminating food limitation bias) and to prevent crabs from feeding only on the largest or most visible food item, as Knudsen (1964) suggested that kelp crabs often respond to movement and use visual cues to locate food. Based on pilot studies, the amount of food provided was enough to avoid food limitation during the feeding experiment. I recorded the blotted wet mass (g) of each macroalgal sample before and after the feeding period. To account for autogenic changes in macroalgal mass over time, control tanks contained macroalgal tissue but no crabs and were run simultaneously to the experiments.

Control and experimental treatments were randomly allocated among the individual tanks. While these controls do not account for the possible influence of fertilization by crab urine, the 12 h feeding period is likely too short for this to strongly influence the results. The feeding experiments took place during both day and night; due to ambient light from outside fixtures, the laboratory was never completely dark. Although it is often suggested that crabs are nocturnal, a careful examination of many natural history and behavioral studies does not provide a clear answer for kelp crabs. Hines (1982) reported little behavioral change in spider crabs during nighttime SCUBA surveys and Zimmer-Faust & Case (1982) reported the kelp crabs endogenous feeding rhythm as unknown. Mesocosm experiments by Hultgren & Stachowicz (2010) indicate that *P. producta* may increase use of kelp habitat at night, but do not provide details on whether this habitat use includes increased feeding on the kelp.

For each experiment, I set up replicate aquaria containing one crab and one blade piece from each macroalgal species (*n* = 7–10; Table 1). Crabs were allowed to feed for 12 h. I analyzed the wet mass consumed (g) for each piece of macroalgae (calculated by subtracting the final mass from the initial mass), and adjusted for the autogenic change in mass by subtracting a randomly-assigned paired control value that contained the same species of macroalgae (Roa, 1992; Duarte et al., 2015). I then used these adjusted values to calculate the Quade’s test statistic (*T*_1_), a non-parametric rank-based test, to test the null hypothesis of no preference, as in Roa (1992). I then used a pairwise post-hoc Quade multiple comparison test with a Bonferroni correction to determine which food types were preferentially consumed.

**Table 1:** Choice feeding experiments on different macroalgal species. Details of macroalgal species comparisons in five separate choice feeding experiments. Parentheses under each algal species report the average starting mass in grams ± the standard error. Sample size, *n*, is equal to the number of crabs used in each “buffet-style” feeding experiment.

Experiment	*n*	Macroalgal species		
		Species 1	Species 2	Species 3
1	7	*Nereocystis luetkeana* (22.8 ± 1.4)	*Alaria marginata* (11.3 ± 0.6)	*Saccharina latissima* (21.8 ± 2.0)
2	9	*N. luetkeana* (24.6 ± 0.9)	*Costaria costata* (31.3 ± 2.4)	*Ulva* sp. (3.0 ± 0.2)
3	10	*N. luetkeana* (17.0 ± 1.7)	*Mazzaella splendens* (8.7 ± 0.7)	*Ulva* sp. (4.0 ± 0.4)
4	10	*N. luetkeana* (17.4 ± 0.8)	*Sargassum muticum* (10.0 ± 0.7)	*Agarum fimbriatum* (11.9 ± 0.4)
5	12	Fresh *N. luetkeana* (17.6 ± 1.4)	Aged *N. luetkeana* (21.3 ± 1.6)	n/a

#### Feeding electivity on aged vs. fresh N. luetkeana

To determine if kelp crabs might be detritivores that choose to eat detached drift kelp, I conducted a choice experiment (*n* = 12 crabs; Table 1; mean crab mass ± SE = 182.1 g ± 23.0 g) using the same methods described above with respect to experiment setup, autogenic controls, time of day and duration, and laboratory conditions on aged and fresh *N. luetkeana.* I collected fresh *N. luetkeana* from kelp beds in the San Juan Channel on the day of the experiment. To simulate detached drift bull kelp, I aged non-reproductive blades in a black-plastic covered outdoor tank with flowing seawater for one week prior to the feeding experiment (Raymond, Lowe & Galloway, 2014). As in the other electivity experiments, each crab was offered an approximately 10 × 30 cm piece of fresh (mean mass ± SE = 17.6 ± 1.4 g) and aged bull kelp (mean mass ± SE = 21.3 g ± 1.6 g) in a choice experiment. I analyzed the changes in mass in the experimental tanks, adjusted by randomly paired control tank changes in mass, using a Wilcoxon signed rank test.

#### Feeding preference on N. luetkeana vs. Lacuna sp.

To determine the trophic tendency of kelp crabs (*n* = 10), I tested the preference of *P. producta* for *N. luetkeana* versus *Lacuna* sp. (<1 cm) in choice and no-choice experiments in the same manner as those described above based on previous laboratory observations of *P. producta* devouring these small snails as well as natural history information suggesting omnivory (Knudsen, 1964). The no-choice experiment represents a block design experiment with crabs as blocks, as each crab was fed kelp and snails separately. I randomly assigned the order of feeding replicates (kelp, snail, or kelp and snail) to each of the 10 crabs. In the choice experiments, I offered crabs an approximately 10 cm × 30 cm piece of kelp (mean mass ± SE = 18.6 g ± 0.9 g) and 20 snails (mean mass ± SE = 0.45 g ± 0.06 g); based on pilot data, these amounts of each food resource represented enough material to ensure that food limitation did not influence the results of the feeding experiment and autogenic changes in these quantities of kelp and snails were minimal during the short time period of the experiment. I analyzed the proportional change in blotted wet mass of kelp and snails (whole, not including shell fragments present at the end of feeding trials) using the Wilcoxon signed rank test for the choice experiments and the Friedman test for the no-choice experiments, treating crabs as blocks (random effect).

#### Crab feeding rates

To examine how feeding rates scale with body size, I used 11 *P. producta* (mass ranging from 80–265 g) and 11 *P. gracilis* (mass ranging from 5–39 g). The crabs were held in flowing seawater tables with unlimited access to fresh *N. luetkeana* to acclimate to laboratory conditions. I starved each crab for 12 h before offering a 10 cm x 30 cm piece of fresh *N. luetkeana* for 12 h (starting mass ranging from 13–21 g). Bull kelp was blotted and weighed before and after the experiment. Crab mass in grams was measured after completion of all feeding experiments. The relationship between kelp mass loss and crab body size was analyzed in R for each crab species using the package lmodel2 to run standardized major axes (SMA) linear regressions, since both kelp mass loss and crab body mass exhibit variability (Legendre, 2014).

### Subtidal caging experiment

To assess the influence of consumers on net tissue gain of juvenile bull kelp (stipe length <30 cm), I designed a subtidal caging field experiment with four treatments. The completely open treatment (*n* = 4) consisted of a small *N. luetkeana* collected from the FHL floating dock with intact holdfast glued directly to a half-size concrete block (15 cm × 20 cm × 20 cm). This treatment left the juvenile bull kelp exposed to any and all consumers (possibly including snails, urchins, and kelp crabs) present in the subtidal environment. The fully enclosed treatment (*n* = 4) consisted of the concrete block, attached kelp, and a wire frame with plastic mesh (with 1 cm × 1 cm openings) on all sides. The partially enclosed treatment (*n* = 4) was a procedural control for the effects of caging. It included the same kind of concrete block with kelp as the open treatment, but had a wire frame covered in plastic mesh on the top and two sides, with the other sides left open. The fully enclosed treatment with a crab (*n* = 4) was identical to the fully enclosed treatment but included one adult *P. producta* (carapace diameter at widest point >10 cm) inside the cage and was supplemented with additional kelp to ensure that the crab survived the entire study period (15 days). Blotted wet mass (mean mass ± SE = 4.31 g ± 0.57 g) and blade length (mean length ± SE = 13.8 cm ± 0.93 cm) of each juvenile kelp was recorded prior to the start of the experiment.

I assembled the concrete blocks in all treatments on land and deployed them from a small boat into a shallow subtidal kelp bed (max depth = 7.6 m) near Point Caution on San Juan Island, Washington, United States (48°33′43.26″N, 123°01′02.33″W). Scuba divers followed the blocks into the water, made sure that all had settled on appropriate horizontal, rocky substrate, and installed the crabs in the appropriate cages. Divers visited the blocks after one week to remove any fouling organisms. Blocks were collected after 15 days. I analyzed the effect of the caging treatment (4 levels) on the change in blotted wet mass and blade length of the juvenile kelp using one-way ANOVA followed by Tukey’s HSD test for pairwise comparisons.

## Results

### Laboratory feeding experiments

**Figure 1:**
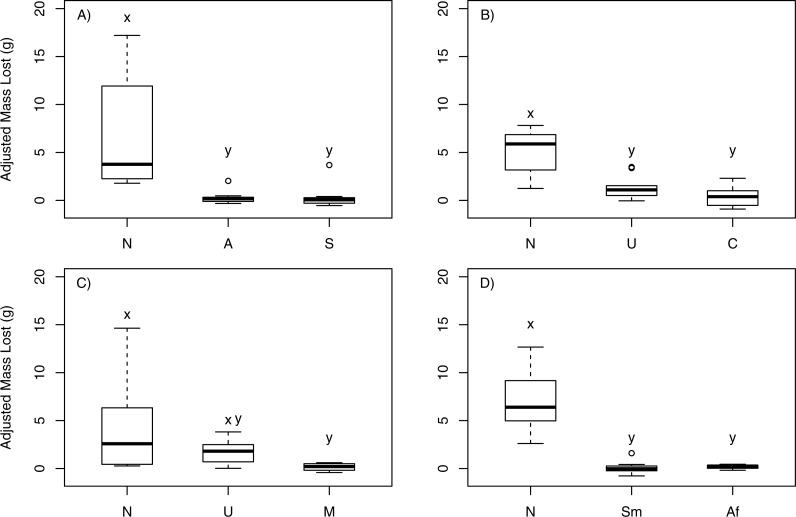
Kelp crab feeding in choice experiments. Change in mass of macroalgae adjusted by change in randomly paired autogenic controls in choice feeding experiments with *P. producta*. Letters correspond to macroalgal species offered: N, *Nereocystis luetkeana*; A, *Alaria marginata*; S, *Saccharina latissima*; C, *Costaria costata*; M, *Mazzaella splendens*; U, *Ulva* sp.; Sm, *Sargassum muticum*; Af, *Agarum fimbriatum*. Box plots show median (dark horizontal line), interquartile range (box), minimum and maximum values (bottom and top “whiskers”), 1st and 3rd quartile (bottom and top of box, respectively), and suspected outliers (dots). Letters indicated statistically significant differences.

In choice feeding experiments, *P. producta* consumed more mass of *N. luetkeana* than of six of the seven other macroalgal species offered (Fig. 1). Among the three kelps tested in the first feeding experiment, crabs showed a statistically significant feeding pattern (*T*_1_ = 8.45 > *F*_0.05;2,12_ = 5.46; *p* = 0.004; Fig. 1A), consuming a much greater mass of *N. luetkeana* (mean mass ± SE = 6.74 g ± 2.36 g) than of the kelps *Alaria marginata* (mean mass ± SE = 0.58 g ± 0.51 g; *p* = 0.01) or *Saccharina latissima* (mean mass ± SE = 0.30 g ± 0.57 g; *p* = 0.01). Crab feeding on *A. marginata* and *S. latissima* was not statistically distinguishable (*p* = 0.99). In the second feeding experiment, crabs also showed a significant pattern (*T*_1_ = 7.62 > *F*_0.05;2,16_ = 4.69; *p* = 0.005; Fig. 1B), consuming a greater mass of *N. luetkeana* (mean mass ± SE = 4.33 g ± 0.88 g) than of the kelp *Costaria costata* (mean mass ± SE = 0.42 g ± 0.36 g; *p* = 0.005) and the green alga *Ulva* sp. (mean mass ± SE = 1.40 g ± 0.30 g; *p* = 0.05). The consumption by *P. producta* of *Ulva* and *Costaria* was statistically similar (*p* = 0.79). When offered *N. luetkeana*, *Ulva* sp. and *Mazzaella splendens* (a red seaweed), consumption by *P. producta* differed (*T*_1_ = 6.26 > *F*_0.05;2,18_ = 4.56; *p* = 0.009; Fig. 1C). The crabs chose to eat very little *M. splendens* (mean mass ± SE = 0.12 g ± 0.07 g; *p* = 0.008) compared to *N. luetkeana* while consuming statistically indistinguishable amounts of *N. luetkeana* (mean mass ± SE = 3.39 g ± 1.53 g) and *Ulva* sp. (mean mass ± SE = 1.84 g ± 0.30 g; *p* = 0.51) as well as *M. splendens* and *Ulva* sp. (*p* = 0.15). When offered a choice between two native kelps (*N. luetkeana*, *Agarum fibriatum*) and the invasive brown alga *S. muticum*, *P. producta* showed significant differences in feeding (*T*_1_ = 14.56 > *F*_0.05;2,18_ = 4.56; *p* < 0.001; Fig. 1D), consuming more *N. luetkeana* (mean mass ± SE = 6.43 g ± 1.03 g) than *A. fimbriatum* (mean mass ± SE = 0.17 g ± 0.05 g; *p* = 0.003) or *S. muticum* (mean mass ± SE = 0.48 g ± 0.15 g); *p* < 0.001). There was no difference in consumption between *S. muticum* and *A. fimbriatum* (*p* = 0.78).

**Figure 2:**
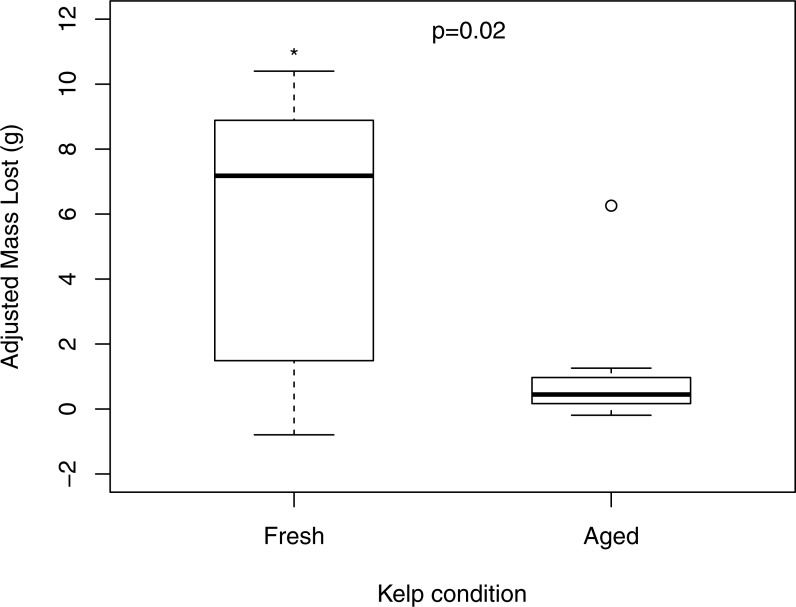
Feeding on fresh and aged bull kelp. Consumption of fresh and aged *Nereocystis luetkeana* by kelp crabs (*Pugettia producta*) in a choice feeding trial (including adjustment from randomly paired autogenic controls). * indicates statistically significant difference in consumption of fresh *N. luetkeana* vs. aged.

**Figure 3:**
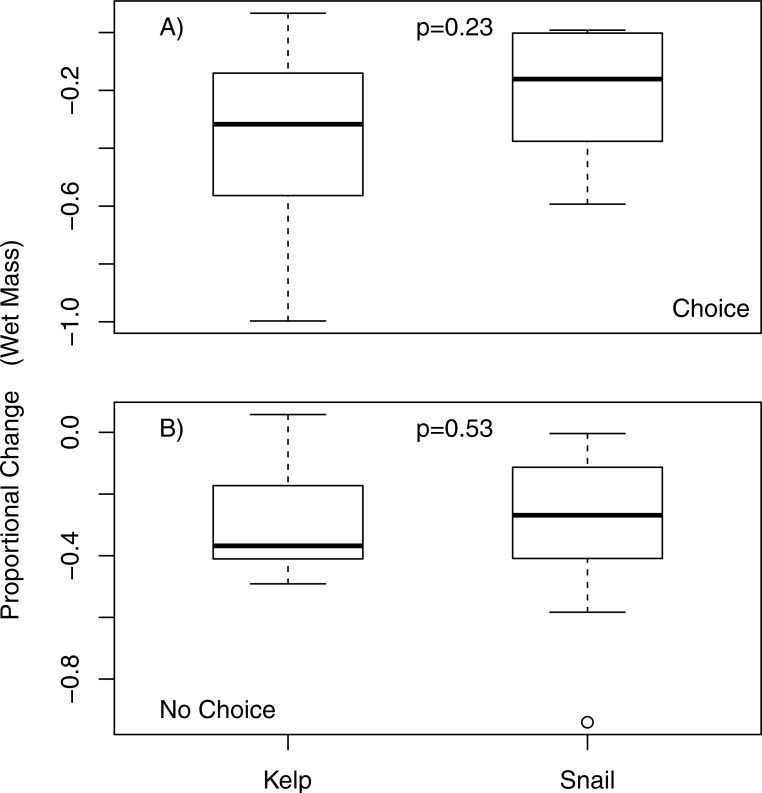
Choice and no choice feeding experiments with kelp and snails. Proportional change (in wet mass) of bull kelp (*N. luetkeana*) and snails (*Lacuna* sp.) in (A) choice and (B) no choice feeding trials; *p*-values from choice and no-choice indicate no significant difference between proportional food consumption in either experiment.

When offered a choice of fresh and aged *N. luetkeana*, *P. producta* consumed both food items, but ate, on average, nearly six times as much fresh *N. luetkeana* (*V* = 50, *p* = 0.02; Fig. 2). I also compared *N. luetkeana* and *Lacuna* sp. as food for *P. producta* in both choice and no-choice feeding experiments. There was no difference between the percentage of mass consumed for kelp and snails in the choice feeding experiments (*V* = 15, *p* = 0.23; Fig. 3A) or in the no-choice feeding experiments (${X}_{1}^{2}=0.4$, *p* = 0.53; Fig. 3B).

**Figure 4:**
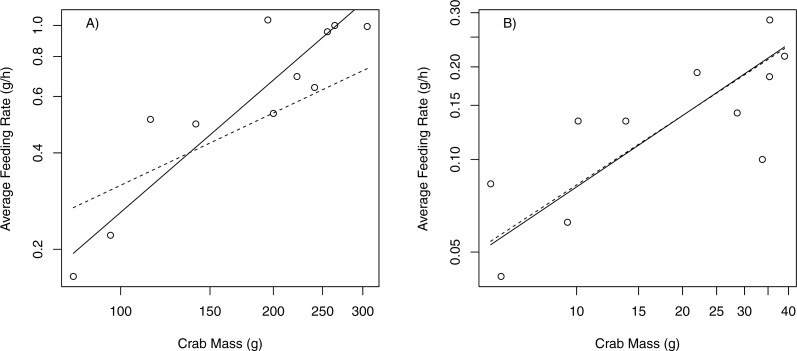
Scaling relationships between feeding rates (g/h) and crab mass (g). Log–log plots showing scaling relationships (SMA regression) between feeding rate (g/h) and crab mass (g) for two species of kelp crabs; (A) *P. producta* shows a noticeable departure from 3∕4 scaling rule; (B) *P. gracilis* shows negative allometry close to the expected 3∕4 rule. Circles and solid line show observed relationship. Dashed line shows the expected slope of 0.75.

When offered only bull kelp, the slope of the relationship between feeding rate (g/h) and body size (g) for P. producta differed significantly from the expected 3/4 scaling rule of metabolic rates (Kiørboe & Hirst, 2014). This indicates that the feeding rate of *P. producta* increases more quickly than crab mass (slope = 1.37; Table 2; Fig. 4A). The feeding rate for *P. gracilis* did not differ from the predicted slope of 0.75 (slope = 0.77; Table 2; Fig. 4B).

**Table 2:** Feeding rate regression analysis in two species of kelp crabs. Relationship between kelp crab feeding rates (g/h) and body mass (g) using standardized major axes (SMA) regression. Observed slopes with confidence intervals that do not overlap 0.75 indicate a significant departure from expected 3∕4 scaling rule.

Species	Predicted slope	Observed slope	95% CI	*y*-intercept	*R*^2^
*P. producta*	0.75	1.37	0.99–1.88	−7.66	0.81
*P. gracilis*	0.75	0.77	0.48–1.21	−4.28	0.61

### Subtidal caging experiment

**Figure 5:**
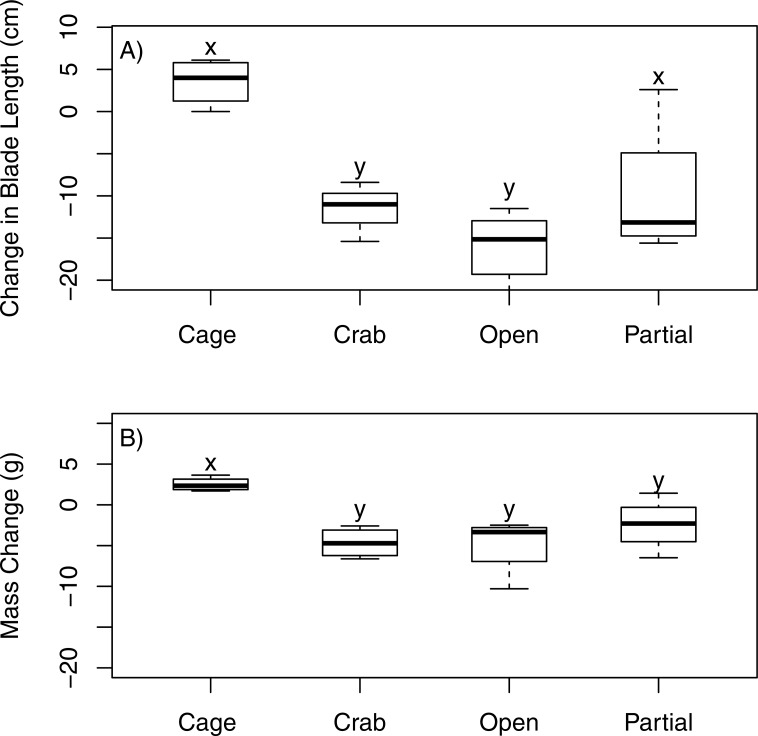
Field exclusion cage experiment. The effect of four experimental treatments on the change in (A) mass and (B) blade length of juvenile *N. luetkeana* in a subtidal field experiment. The “cage” treatment was fully enclosed to protect the individual kelp from all large grazers; the “crab” treatment was fully enclosed with one adult *P. producta* included inside; the “open” treatment offered no protection to attached kelp; the “partial” treatment was a procedural control that included caging material over the top and two sides but left the other two sides open. Letters indicated statistically significant differences.

Juvenile bull kelp mass varied significantly among treatments (ANOVA, *F*_3,12_ = 6.711, *p* = 0.007). *Nereocystis luetkeana* (<30 cm stipe length) increased in mass by 77% over 15 days in the fully enclosed (caged) treatment but suffered extreme loss of tissue in the treatments that were open (−95%), partially enclosed (−55%), and fully enclosed with a crab (−98%) treatments (Fig. 5A). Mass of *N. luetkeana* in the fully enclosed treatment differed statistically from the mass in the treatments that were open and fully enclosed with a crab (Tukey’s HSD, *p* < 0.01); the difference between the fully caged and partially caged treatment approached statistical significance (Tukey’s HSD, *p* = 0.09).

Similarly, juvenile bull kelp length varied among treatments (ANOVA, *F*_3,12_ = 10.4, *p* = 0.002). Only the juvenile bull kelp in the fully enclosed (caged) treatment showed a positive change in blade length (33.15%). Blade tissue was completely lost (−100%) in the open and fully enclosed with a crab treatments and was greatly decreased in the partially enclosed (−70%) treatment (Fig. 5B). Blade length of *N. luetkeana* in the fully enclosed treatment was statistically different from the other treatments (Tukey’s HSD, *p* < 0.02).

## Discussion

Herbivory by Northern kelp crabs (*P. producta*) may play a larger role in structuring bull kelp forests than previously suspected, as kelp crabs elect to eat fresh bull kelp over other macroalgae and also consume a greater quantity of bull kelp in relation to body mass than the 3∕4 metabolic scaling rule would suggest. However, kelp crabs also readily consume snails, indicating some tendency toward omnivory. In the field, protecting juvenile bull kelp from large consumers led to net tissue increases, further suggesting some level of top-down pressure from large consumers.

I found that kelp crabs elected to feed on bull kelp over other co-occuring macroalgae in the laboratory. The attributes that reduce kelp crab feeding on other kelps may include morphology or chemistry; however, no clear role for either factor emerges from comparisons across the kelp species tested in this study. Both *S. latissima* and *C. costata* are thick bladed with bullations, but *A. marginata* “wings” (blade) on either side of the midrib have a thinner morphology similar to bull kelp but were nevertheless consumed less than *N. luetkeana*. *A. marginata*, *C. costata,*, and *N. luetkeana* share low levels of phlorotannins (less than 1% dry mass for each) in their blades, with some environmental variability in concentration (Van Alstyne et al., 1999; Van Alstyne, Whitman & Ehlig, 2001). Urchins do not prefer *C. costata,* although other invertebrates will consume it (Van Alstyne, Ehlig & Whitman, 1999). Some species of the green seaweed *Ulva* have activated chemical defenses but also favorable nutritional characteristics (high nitrogen), which might account for reduced, but not absent, consumption by kelp crabs (Van Alstyne, Pelletreau & Kirby, 2009). *Mazzaella splendens* was also not preferentially consumed by generalist herbivore snails (Van Alstyne, Pelletreau & Kirby, 2009) This red seaweed appears iridescent due to its cuticle structure, which might function as a visual or mechanical deterrent to feeding (Gerwick & Lang, 1977). Unlike *L. vincta*, which has been shown to live on and consume invasive *S. muticum* (Britton-Simmons et al., 2010), Northern Kelp crabs did not choose to consume *S. muticum* in laboratory feeding experiments, indicating that this invasive seaweed is not a food substitute for native bull kelp for all local consumers. The mechanisms underlying diet choice by kelp crabs require additional investigation.

Kelp crab electivity for bull kelp aligns with diets of other marine macroalgae consumers. Previous laboratory and field experiments showed that *N. luetkeana* was frequently the preferred brown algal food of a snail, *Tegula funebralis* (Steinberg, 1985). Sea urchins have also been shown to prefer bull kelp over other macroalgae and displayed high absorption efficiencies and growth rates in laboratory feeding experiments (Vadas, 1977). For herbivorous amphipods, kelp is most palatable as a food source when the carbon to nitrogen ratio is neither too high nor too low (Norderhaug, Nygaard & Fredriksen, 2006).

However, the feeding rates by kelp crabs that I measured are large, both in absolute and body size specific terms compared with other kelp grazers present in the kelp beds of the Salish Sea. Northern and graceful kelp crabs can consume large quantities of kelp; on average, *P. producta* consumed *N. luetkeana* at 0.65 g/h, while *P. gracilis* consumed at 0.14 g/h, consistent with generally smaller body size. These rates correspond to 8% and 20% of body mass per day, respectively. In contrast, green urchins (*S. droebachiensis*) can consume kelp at a rate of 0.052 g/h and red sea urchins (*S. franciscanus*) consume 0.17 g/h (Vadas, 1977), rates that correspond to about 2% of their respective body masses per day. Additionally, the feeding rate of *P. producta* on bull kelp that exceeds the 3∕4 rule of metabolic scaling indicates that in this species larger individuals can be disproportionately more voracious consumers of bull kelp. Bull kelp would be particularly vulnerable to herbivory as a juvenile (stipe length <30 cm), which could be entirely consumed by a kelp crab within one to a few days.

Although these results suggest kelp crabs exert underappreciated control of the dynamics of this annual kelp species through direct grazing, Northern kelp crabs, *P. producta,* may not be as exclusively herbivorous as previously reported (e.g., Knudsen, 1964; Kozloff, 1983). While they consumed fresh macroalgae (herbivory), they also ate small snails (omnivory), and aged bull kelp (detritivory) in laboratory feeding trials. It was unexpected that crabs would maintain their feeding rate on both snails and bull kelp, regardless of whether these species were offered singly or in combination with the other resource. However, it is worth noting that the total mass of snails eaten was small even if the proportional consumption was similar. This may result from the long handling times observed in pilot studies. Crabs in choice treatments were offered ∼10 g of kelp and ∼0.1 g of snails. Nevertheless, kelp crabs that consume small snails in the field might indirectly facilitate bull kelp, in addition to direct negative effects, by removing another grazer that feeds on kelp and causes damage that weakens the thallus. *P. producta* disproportionately uses fresh over aged kelp, consuming an average of five times more fresh bull kelp, indicating that they are unlikely to be detritivores when live kelp is available. This differs from red urchin feeding in the San Juan Channel (Britton-Simmons, Foley & Okamoto, 2009). This difference in electivity indicates that kelp crabs are likely to actively consume the kelp on which they live instead of passively consuming detached drift kelp present in the subtidal environment with potentially greater implications for kelp population dynamics.

The result that juvenile bull kelp completely protected from large consumers increased net mass and blade length more than those exposed to herbivory in the subtidal experiment indicates that some level of top-down control of bull kelp populations exists in this system. While the change in mass of kelp in the partially open cages was not statistically significant from the other treatments, the median change was similar to the other treatments with some level of consumer access. The high variability present in the partially open treatment may be due to consumers having more difficulty accessing and consuming the kelp in this treatment. The magnitude of damage experienced by kelp in completely open treatments was similar to the level of damage to kelp enclosed in a cage with a crab, which suggests that kelp crabs could be a particularly influential cause of failed kelp recruitment, although other herbivores (including red and green urchins and herbivorous snails) may also have contributed. The mesh on the cages had holes large enough (1 cm ×  1 cm) to allow small snails to enter, potentially to consume the kelp or be consumed by the enclosed crab. However, it seems unlikely that snails alone could cause the level of damage exhibited by the exposed juvenile kelp, and the increase in net mass as well as blade length of the fully enclosed individuals indicates that kelp in open treatments experienced herbivory from some larger consumer than a snail. Because bull kelp is an annual species and must complete its entire life cycle each year, the effect of destructive grazing by herbivores could be especially dangerous during the small, vulnerable period; laboratory feeding studies have suggested that *L. vincta* prefers juvenile *N. luetkeana* to adult tissue (Chenelot & Konar, 2007), and this may hold true for other kelp consumers as well, including kelp crabs. Herbivore-inflicted damage to the stipes of small bull kelp could also make them more vulnerable to breakage from abiotic forces, increasing indirect losses on top of direct consumption.

Kelp crab feeding in the Salish Sea near the San Juan Islands has not produced barrens like those created by high densities of sea urchins in other geographical regions (Scheibling, Hennigar & Balch, 1999; Steneck et al., 2002), possibly due to the high levels of diversity in terms of potential food resources (both macroalgal and invertebrate) as well as rich nutrient and detrital inputs in this system that limit strong top-down control. However, the combined results of these laboratory feeding and subtidal field experiments suggest that kelp crabs may be one of a number of kelp consumers that negatively impact the growth of bull kelp. While urchins and snails likely cause some of the observed damage to bull kelp in the nearshore subtidal, my laboratory and field experiments clearly show that Northern Kelp crabs elect to eat *N. luetkeana* over most other macroalgae, can eat even larger quantities than might be expected for a given crab’s mass, and cause damage of great enough magnitude to decimate small bull kelp. For these reasons, Northern Kelp crab (*P. producta*) may play a previously unrecognized role as an important consumer influencing the dynamics of the annual bull kelp. Future work to quantify kelp crab abundance in subtidal kelp forest habitats in the Salish Sea will help to further illuminate the level of top-down control that kelp crabs might exert on bull kelp populations during different life stages.

##  Supplemental Information

10.7717/peerj.3372/supp-1 Supplemental Information 1 Raw field data Raw data used for analysis of subtidal cage experiment, Fig. 5.

10.7717/peerj.3372/supp-2 Supplemental Information 2 Feeding trial raw data Raw data used for analysis, Figs. 1 and 2.

10.7717/peerj.3372/supp-3 Supplemental Information 3 Feeding trial controls Raw control data used for analysis, Figs. 1 and 2.

10.7717/peerj.3372/supp-4 Supplemental Information 4 Kelp and snails feeding data Raw data for kelp vs. snail (choice and no choice) experiment analysis, Fig. 3.

10.7717/peerj.3372/supp-5 Supplemental Information 5 Raw data for kelp crab feeding rate scaling Raw data used for analysis of kelp crab feeding rate scaling, Fig. 4.

## Additional Information and Declarations

### Data Availability

The following information was supplied regarding data availability: The raw data has been supplied as a Supplementary File.
